# Biphasic Properties of PVAH (Polyvinyl Alcohol Hydrogel) Reflecting Biomechanical Behavior of the Nucleus Pulposus of the Human Intervertebral Disc

**DOI:** 10.3390/ma15031125

**Published:** 2022-01-31

**Authors:** Minhyeok Heo, Seonghun Park

**Affiliations:** School of Mechanical Engineering, Pusan National University, Busan 46241, Korea; hmh555@hanmail.net

**Keywords:** PVAH, lumbar spine, biphasic continuum model, nucleus pulposus, Holmes–Mow model

## Abstract

PVAH is a mixture of solid and fluid, but its mechanical behavior has usually been described using solid material models. The purpose of this study was to obtain material properties that can reflect the mechanical behavior of polyvinyl alcohol hydrogel (PVAH) using finite element analysis, a biphasic continuum model, and to optimize the composition ratio of PVAH to replace the nucleus pulposus (NP) of the human intervertebral disc. Six types of PVAH specimens (3, 5, 7, 10, 15, 20 wt%) were prepared, then unconfined compression experiments were performed to acquire their material properties using the Holmes–Mow biphasic model. With an increasing weight percentage of PVA in PVAH, the Young’s modulus increased while the permeability parameter decreased. The Young’s modulus and permeability parameter were similar to those of the NP at 15 wt% and 20 wt%. The range of motion, facet joint force, and NP pressures measured from dynamic motional analysis of the lumbar segments with the NP model also exhibited similar values to those with 15~20 wt% PVAH models. Considering the structural stability and pain of the lumbar segments, it appears that 20 wt% PVAH is most suitable for replacing the NP.

## 1. Introduction

Low back pain is the most common disease in modern society. Degenerative intervertebral discs (IVDs) account for more than 75% of the causes of low back pain [1]. The nucleus pulposus (NP) of the IVD applies intradiscal pressure to the annulus fibrosus (AF) [2]. When the NP of the degenerated IVD has reduced water content, there is abnormal stress on the AF [2,3,4]; this condition is treated by using a replacement for the NP [5]. NP replacement can reduce pain while restoring spine mobility and delay IVD degeneration [6,7,8,9,10,11]. Moreover, this treatment has the advantage of preserving AF with minimally invasive surgery.

Some studies have reported that the NP can be modeled as an incompressible fluid and an isotropic solid with a porous structure [4,12,13,14,15,16], while others have shown that the NP exhibits properties of both solid and fluid materials simultaneously [4,17,18]. Although various studies have been conducted to explore the material models of the NP by performing experimental and analytical studies [14,19,20], these material models do not accurately reflect the biomechanical behavior of the NP and that of the entire IVD with the NP [21]. Currently, a biphasic continuum model that implements anisotropy, nonlinearity, and inhomogeneity is widely used to model the NP and simulate its biomechanical behavior [22,23,24,25].

Hydrogels are crosslinked polymer networks with high water content. Polyvinyl alcohol (PVA) is a synthetic water-soluble polymer with low toxicity, excellent mechanical strength, and high biocompatibility. PVA hydrogel (PVAH) has been used in various applications, including arterial phantoms [26], heart valves [27], corneal implants [28], and artificial cartilage [29]. PVAH exhibits a large deformation for relatively small mechanical loads and time-dependent viscoelastic behavior, such as rubber materials. One of the applications of this material is the treatment of degenerative IVDs [30,31]. To investigate the mechanical behavior of PVAH, various tests, including unconfined and confined compression tests, have been performed, and PVAH has been described using hyperelastic and viscoelastic material models [1,32,33]. However, because PVAH consists of a porous solid (PVA) and a fluid (body fluid) that fills it, there are limitations in describing its mechanical behavior with material models that only simulate its solid part.

Therefore, the purpose of this study was to obtain the material properties of PVAH, and then optimize its composition ratio such that it can reflect the mechanical behavior of the NP, using finite element analysis (FEA) and a biphasic continuum model. To this end, PVAH specimens with various composition ratios of PVA and PBS were first generated to experimentally measure the material properties used for the NP in the finite element (FE) model of the lumbar spine. Next, in the lumbar spine model with PVAH to replace the NP, the range of motion (ROM) and facet joint force (FJF) for flexion, extension, lateral bending, and axial torsion were analyzed to select the optimal PVAH composition ratio.

## 2. Materials and Methods

### 2.1. PVAH Specimen Preparation

In Jordan’s unconfined test results, the maximum compressive stress of human nucleus pulposus was about 4 kPa [33], and this value was less than the maximum compressive stress of 10% PVA in Kobayasi’s study [34]. Therefore, in this study, PVAH having a composition between 3 wt% and 20 wt%, polyvinyl alcohol was selected as follows:

Phosphate-buffered saline (PBS, ×10 Concentrate, Sigma-Aldrich, St. Louis, MO, USA) was diluted ten times with distilled water. PVA (polyvinyl alcohol, molecular weight ~89,000–98,000 g/mol, 99+% hydrolyzed, Sigma-Aldrich, St. Louis, MO, USA) with a molecular weight (Mw) of 8.9 × 10^4^ ~ 9.8 × 10^4^ was prepared. Six types of PVAH specimens (PVAH1: 3 wt% PVA and 97 wt% PBS; PVAH2: 5 wt% PVA and 95 wt% PBS; PVAH3: 7 wt% PVA and 93 wt% PBS; PVAH4: 10wt% PVA, 90 wt% PBS; PVAH5: 15 wt% PVA, 85 wt% PBS; PVAH6: 20 wt% PVA, 80 wt% PBS) were prepared.

**Figure 1:**
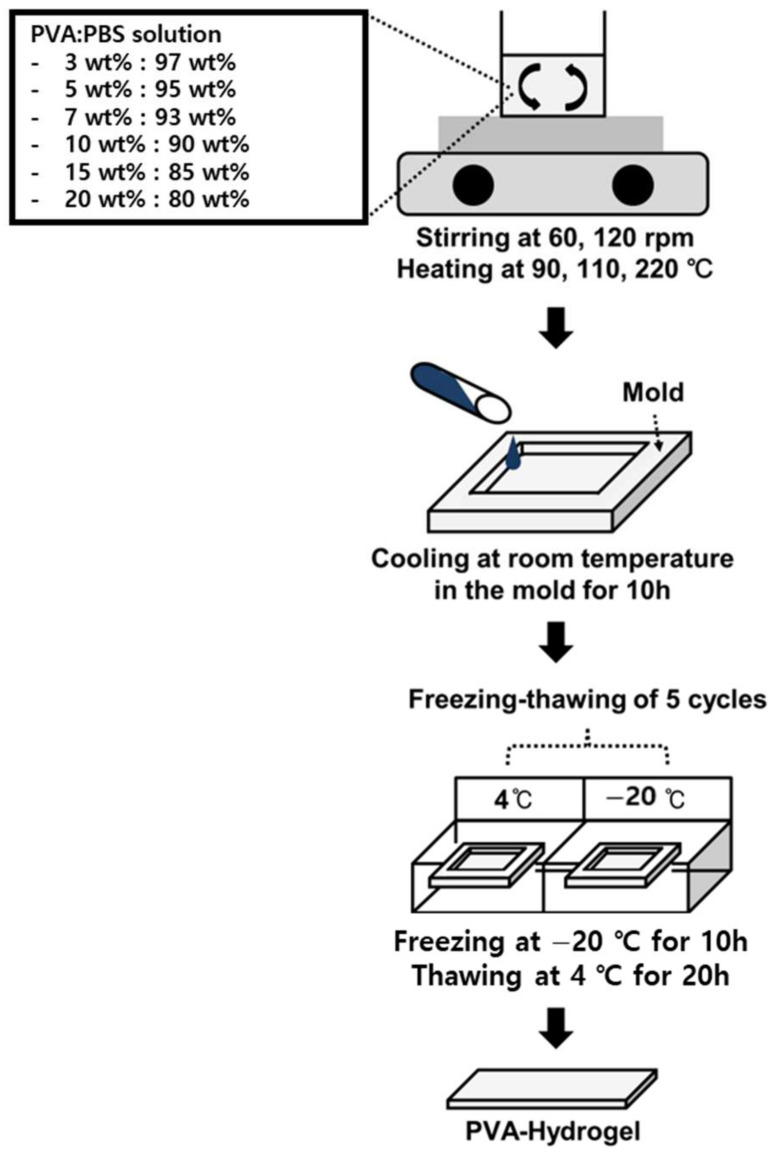
Schematic diagram of PVAH (Polyvinyl Alcohol Hydrogel) production process.

When preparing the specimen, the PVA:PBS solution was stirred and heated for 2 h. The temperature was gradually raised to 160 °C by stirring the specimen at a speed of 120 rpm for 1 h and 30 min. The temperature was then increased slowly to 220 °C for 30 min by stirring at 60 rpm to prevent excessive evaporation due to a sudden phase change. Finally, to remove bubbles generated during stirring and heating, the temperature was reduced to 100 °C, and the heating conditions were maintained for approximately 10 min, after which the stirring was stopped. The prepared PVAH specimen was poured into a self-made mold and stored at room temperature for 10 h for bubble removal and stabilization. The samples were then frozen at −20 °C for 10 h and thawed at 3 °C for 20 h. This process was repeated five times [35]. The completed PVAH specimen was placed in a 1× PBS solution and stored in a refrigerator (Figure 1).

### 2.2. Holmes–Mow Model

The Holmes–Mow material model was used to represent the non-fibrillar solid matrix of soft tissues, including articular cartilage and annulus fibrosus [20,36,37,38,39]. The Holmes–Mow model is expressed in terms of elastic solids and hydraulic permeability.

Here, elastic solids can be formulated constitutively by the coupled hyperelastic strain–energy function (Ψ), as described by

(1) $$\Psi \left(I_{1},I_{2},J\right)=\frac{1}{2}c\left(e^{Q}-1\right)$$

where $I_{1}$ and $I_{2}$ are the first and second invariants of the right Cauchy-Green tensor, and J is the Jacobian of the deformation. The exponential strain components related to function Q and material parameter c are formulated by:

(2) $$Q=\frac{\beta }{\lambda +2\mu }\left[\left(2\mu -\lambda \right)\left(I_{2}-3\right)+\lambda \left(I_{2}-3\right)-\left(\lambda +2\mu \right)ln\left(J^{2}\right)\right]$$

(3) $$c=\frac{\lambda +2\mu }{2\beta }$$

where β is the exponential stiffening coefficient that accounts for the nonlinearity of the matrix stress–strain response. The material parameters λ and μ can be expressed by Young’s modulus (E) and Poisson’s ratio (υ) as follows:

(4) $$\lambda =E/\left(1+\upsilon \right)\left(1-2\upsilon \right)\;$$

(5) $$\mu =E/\left(2\left(1+\upsilon \right)\right)$$

In biological tissues, compressive forces reduce the size and change the shape of the pores within a solid matrix. This leads to a nonlinear increase in the frictional interaction and momentum exchange between the fluid and solid. To explain this, Holmes–Mow’s hydraulic permeability is employed as follows:

(6) $$k\left(J\right)=k_{0}{\left(\frac{J-\varphi_{0}}{1-\varphi_{0}}\right)}^{\alpha }e^{\frac{1}{2}M\left(J^{2}-1\right)}$$

where J is the Jacobian of the deformation, k is the hydraulic permeability in the current state, $k_{0}$ is the permeability in the reference state, $\varphi_{0}$ is the solid volume fraction in the reference state, M is the exponential strain-dependent coefficient, and α is the power law exponent.

### 2.3. Unconfined Compression Experiments

**Figure 2:**
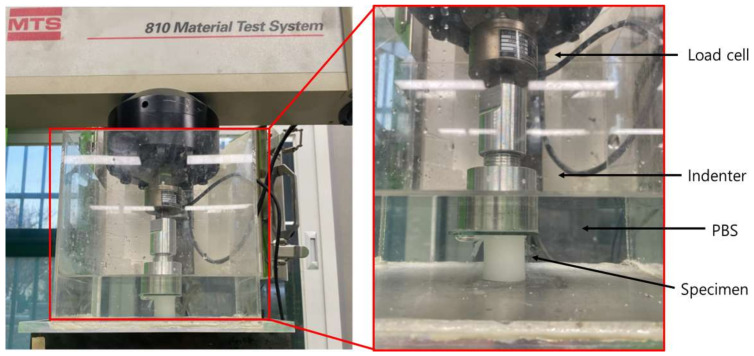
Material testing apparatus.

Unconfined compression experiments were performed using a dynamic material tester (Model 809, MTS Systems, Eden Prairie, MN, USA) with a 500 N load cell. A transparent acrylic chamber with a height of 10 cm was used to place the PVAH specimens with PBS solution (Figure 2). For the unconfined compression experiments, 30 PVAH specimens (five specimens × six types of PVAH: five specimens for each type of PVAH), 30 mm in diameter and 30 mm in height, were prepared and loaded with a flat indenter attached to the load cell. The indenter was initially lowered to contact the specimen. Compressive loads were then applied to the specimen up to 25% strain at a rate of 5% per minute. The 5% strain was maintained for 3 h to observe the stress relaxation.

The experimental results were curve-fitted to the Holmes–Mow model using the optimization algorithm of FEBio software (open-source, version 1.5.0, Weiss Lab—University of Utah, and Ateshian Lab—Columbia University, USA), and the elastic parameters in the model, such as Young’s modulus (E), Poisson’s ratio (υ), nonlinear permeability parameters $k_{0}$ and exponential coefficients β and M, were extracted.

### 2.4. Range of Motion (ROM), Facet Joint Force (FJF), and Nucleus Pulposus (NP) Pressures

The lumbar finite element (FE) model was generated using lumbar shape data from the Human Anatomy Model (Viewpoint Datalabs, Orem, Utah, USA). The lumbar vertebrae are composed of cancellous bone, cortical bone, posterior element, and endplate. The endplate was modeled as a bony endplate (BEP) and cartilage endplate (CEP). A linear elastic model was adopted for cancellous bone, cortical bone, the posterior element, and BEP, while the Holmes–Mow model was used for CEP. The articular cartilage of the facet joint was modeled to be attached to the upper and lower posterior elements, and the initial gap between the upper and lower cartilages was set to 0.5 mm. It was assumed that there was no friction between the upper and lower cartilage of the facet joint [40,41].

The intervertebral disc (IVD) was modeled with annulus fibrosis (AF) and nucleus pulposus (NP) [42,43]. The AF was modeled as two layers of the outer annulus fibrosus (OAF) and inner annulus fibrosus (IAF) [44]. The solid matrix of the AF was modeled using the Mooney–Rivlin model, whereas its fluid was modeled using the Holmes–Mow hydraulic permeability. The AF fiber was simulated by adding a fiber-pow-linear model to the Mooney–Rivlin model of the solid matrix. The fluid and solid of the NP were simulated using the Holmes–Mow model (Table 1).

**Figure 3:**
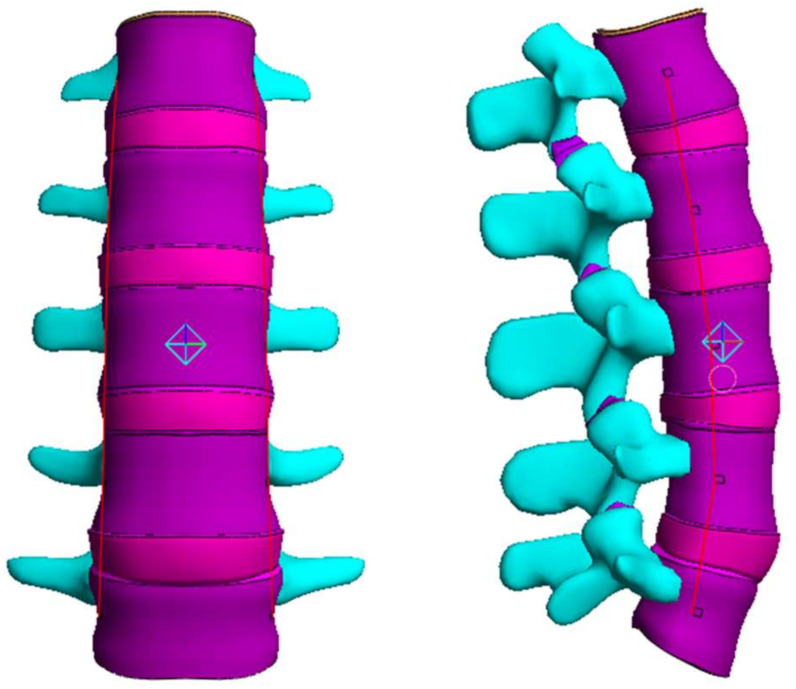
Finite element (FE) model of the lumbar segments.

Lastly, the lumbar ligaments, including the anterior longitudinal ligament (ALL), posterior longitudinal ligament (PLL), transverse ligament (TL), interspinous ligament (ISL), supraspinous ligament (SSL), capsular ligament (CL), and ligamentum flavum (LF), were modeled to be attached to the lumbar spine based on anatomical information. The behavior of the ligament was simulated using a spring element that only resists tension (Figure 3) [49,50,51].

To validate the normal lumbar FE model, the ROMs, FJFs, and NP pressures (maximum stresses of the IAF and OAF) were compared with analytical and experimental literature findings for flexion, extension, lateral bending, and axial torsion motions. To compare the literature findings and the current results, the loading conditions shown in Table 2 were applied to the upper surface of the L1 lumbar vertebrae, while the lower surface of the L5 lumbar vertebra was fixed so as not to cause displacement in any direction [52,53,54]. For finite element (FE) analysis of PVAH composition optimization, the ROM and FJF for flexion, extension, lateral bending, and axial torsion motions were calculated under the same loading conditions as above (Table 2). Here, the material properties of six different types of PVAH specimens were experimentally obtained from the unconfined compression experiments and subsequent curve fitting and then used in the Holmes–Mow model of the NP. The results of the ROMs and FJFs were compared with the analytical and experimental results of the normal lumbar FE model with the normal NP.

## 3. Results

### 3.1. Unconfined Compression Experiments

**Figure 4:**
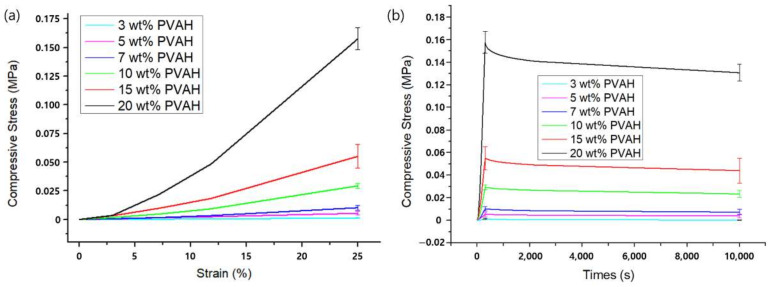
(**a**) Stress–strain and (**b**) stress–relaxation curves of six types of typical PVAH specimens with different composition ratios of PVA and PBS obtained from unconfined compression experiments.

**Figure 5:**
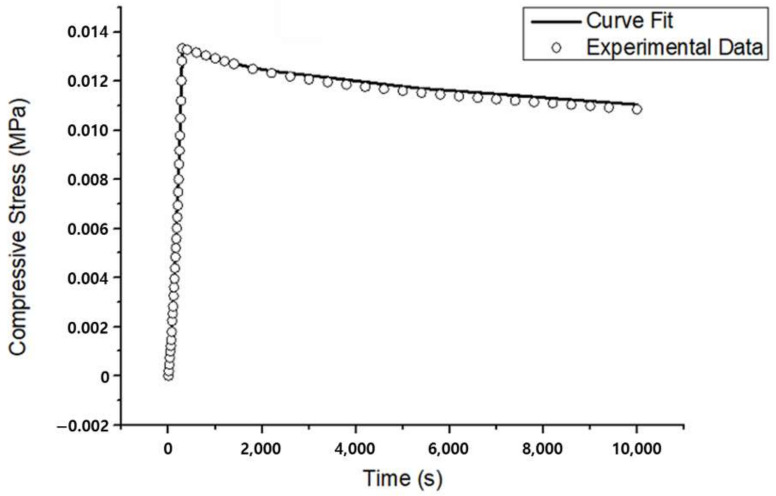
Stress relaxation curve as a function of time and corresponding curve fit $\left(R^{2}=0.999\right)$ for a typical PVAH specimen with 7 wt% PVA and 93 wt% PBS.

In the unconfined compression experiments, all six types of PVAH exhibited a nonlinear stress–strain response curve (Figure 4). The maximum compressive stress of 20 wt% PVAH was greater than those of 3 wt% PVAH by about 143 times. The stress–relaxation rate was about 4.27 times greater for 3 wt% PVAH compared to 20 wt% PVAH. As the water content of PVAH increased, the maximum compressive stress decreased, whereas the amount of stress relaxation behavior increased. The elastic and permeability parameters in the Holmes–Mow model to represent PVAH in FEA were acquired by curve fitting of these results using the optimization algorithm of FEBio (Figure 5). It was found that the Holmes–Mow model could successfully fit the experimental stress–strain response data with errors less than 1 × 10^−6^. With an increasing PVA content of PVAH, the Young’s modulus (E) increased, while the nonlinear permeability parameter ($k_{0}$) decreased (Table 3).

The E and $k_{0}$ of the NP were between those of 15 wt% PVAH and 20 wt% PVAH. The E of the NP was ~39% higher than that of 15 wt% PVAH and ~72% lower than that of 20 wt% PVAH, while $k_{0}$ of the NP was ~295% lower than that of 15 wt% PVAH and ~37% higher than that of 20 wt% PVAH.

### 3.2. Range of Motion (ROM), Facet Joint Force (FJF), and Nucleus Pulposus (NP) Pressures

**Figure 6:**
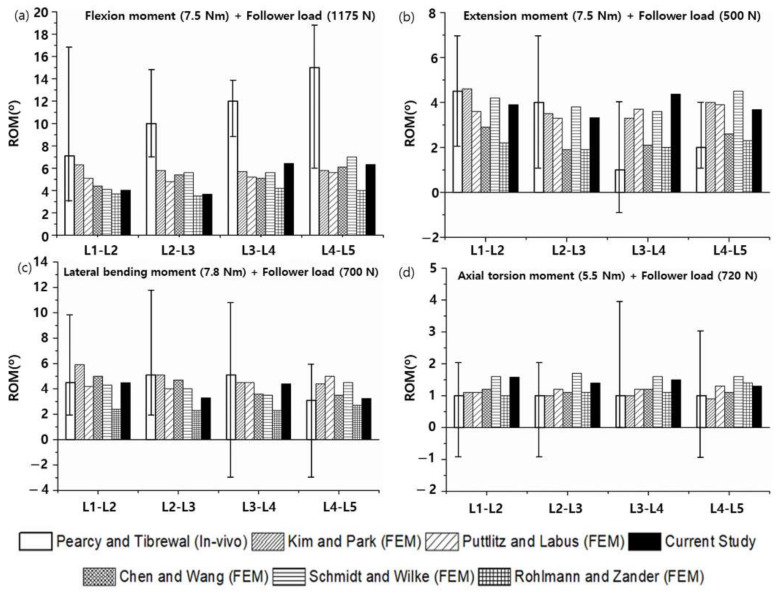
Range of motions (ROMs) of the healthy lumbar spine segments measured under a combination of (**a**) flexion moment (7.5 N∙m) and follower load (1175 N), (**b**) extension moment (7.5 N∙m) and follower load (500 N), (**c**) lateral bending moment (7.8 N∙m) and follower load (700 N), and (**d**) axial rotation moment (5.5 N∙m) and follower load (720 N) (the white bars represent in vivo experimental results of the ROM for the L4–L5 segment, and other bars represent previous and current findings obtained from finite element analyses).

**Figure 7:**
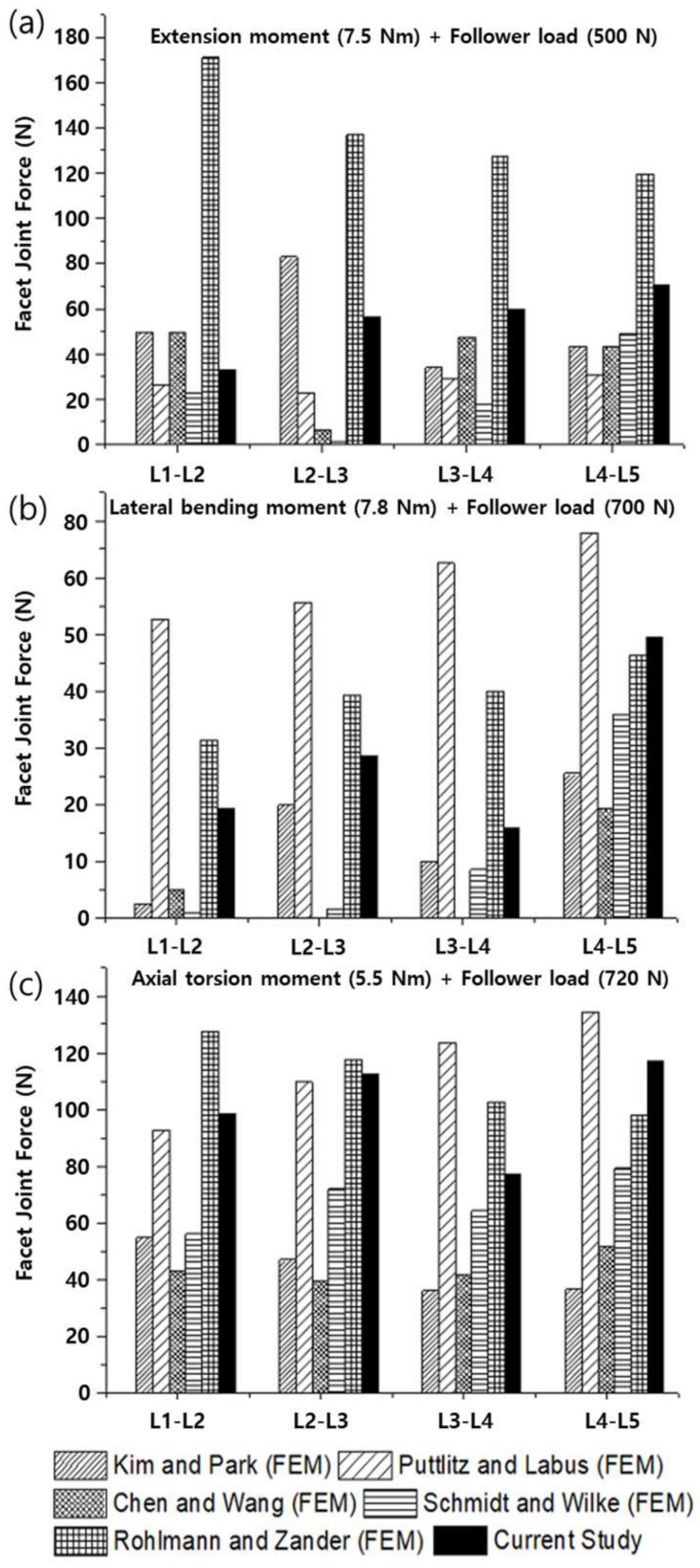
Facet joint forces (FJFs) of the healthy lumbar spine segments measured under a combination of (**a**) extension moment (7.5 N∙m) and follower load (500 N), (**b**) lateral bending moment (7.8 N∙m) and follower load (700 N), and (**c**) axial rotation moment (5.5 N∙m) and follower load (720 N).

**Figure 8:**
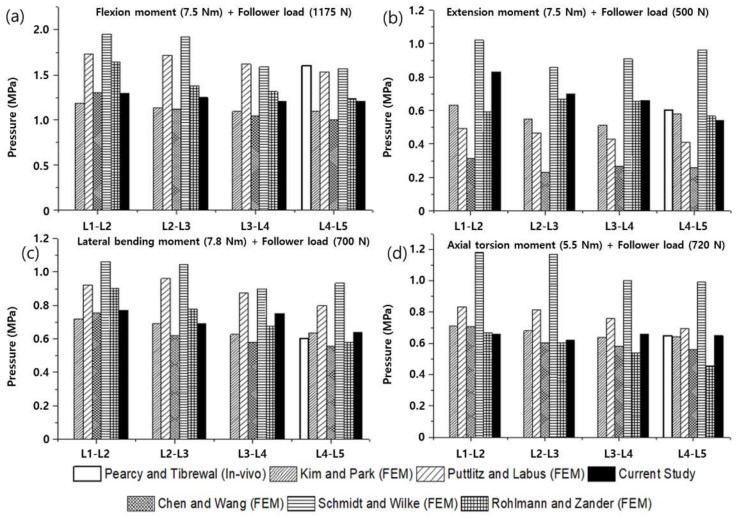
Nucleus pulposus (NP) pressures of the healthy lumbar spine segments measured under a combination of (**a**) flexion moment (7.5 N∙m) and follower load (1175 N), (**b**) extension moment (7.5 N∙m) and follower load (500 N), (**c**) lateral bending moment (7.8 N∙m) and follower load (700 N), and (**d**) axial rotation moment (5.5 N∙m) and follower load (720 N) (the white bars represent in vivo experimental results of the NP pressures for the L4–L5 segment, and other bars represent previous and current findings obtained from finite element analyses).

To verify the modeled lumbar spine segments, the ROM for flexion, extension, lateral bending, and axis torsion motions measured under a combination of moments and follower loads were compared with the results of previous studies. The present results were consistent with the ROMs measured in previous in vivo and analytical studies (Figure 6) [55,56,57,58]. Likewise, the FJFs and NP pressures for flexion, extension, lateral bending, and axis torsion motions were consistent with previous findings measured from lumbar spine finite element (FE) models (Figure 7 and Figure 8) [55,56,57,58].

**Figure 9:**
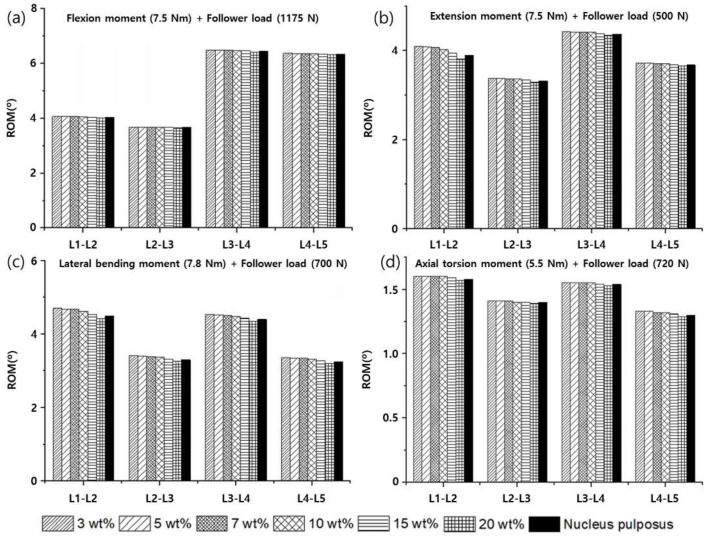
Range of motions (ROMs) of the lumbar spine segments with PVAH to replace the NPs that were measured under a combination of (**a**) flexion moment (7.5 N∙m) and follower load (1175 N), (**b**) extension moment (7.5 N∙m) and follower load (500 N), (**c**) lateral bending moment (7.8 N∙m) and follower load (700 N), and (**d**) axial rotation moment (5.5 N∙m) and follower load (720 N).

**Figure 10:**
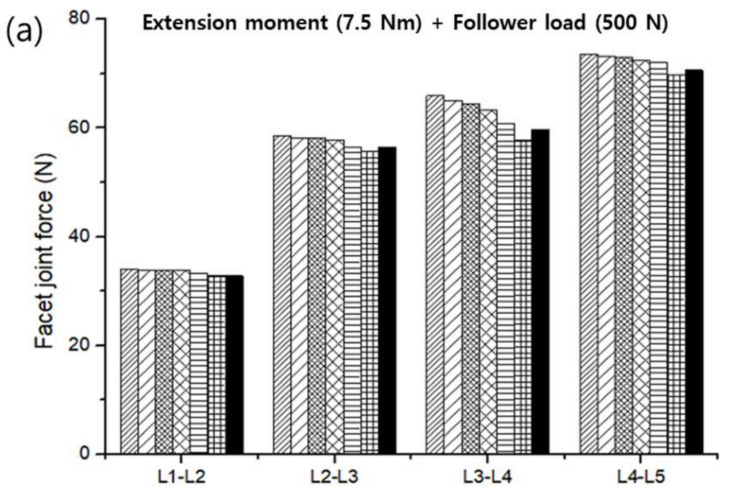

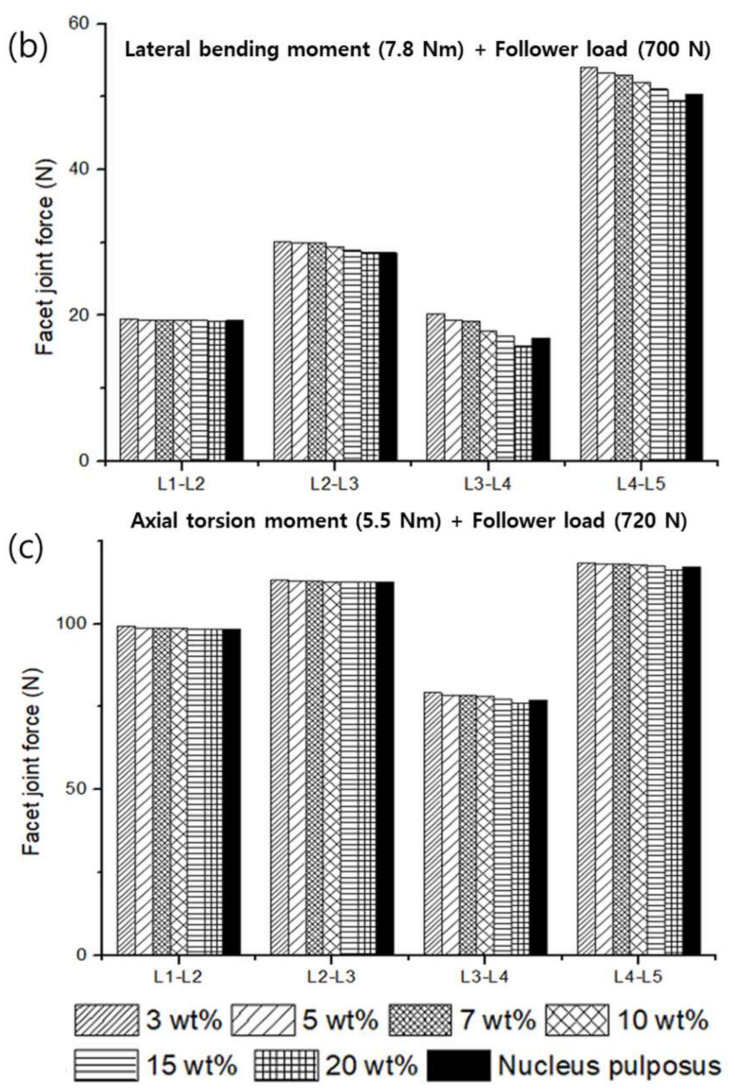
Facet joint forces (FJFs) of lumbar spine segments with PVAH to replace the NPs that were measured under a combination of (**a**) extension moment (7.5 N∙m) and follower load (500 N), (**b**) lateral bending moment (7.8 N∙m) and follower load (700 N), and (**c**) axial rotation moment (5.5 N∙m) and follower load (720 N).

**Figure 11:**
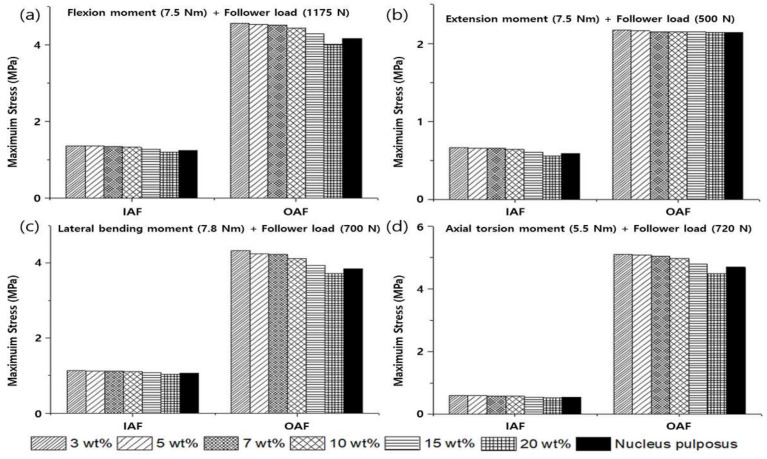
Maximum stresses for the inner annulus fibrosus (IAF) and the outer annulus fibrosus (OAF) of the lumbar spine segments with PVAH to replace the NPs that were measured under a combination of (**a**) flexion moment (7.5 N∙m) and follower load (1175 N), (**b**) extension moment (7.5 N∙m) and follower load (500 N), (**c**) lateral bending moment (7.8 N∙m) and follower load (700 N), and (**d**) axial rotation moment (5.5 N∙m) and follower load (720 N) (each colored bar represents the average value of maximum stresses calculated in the L1–L2, L2–L3, L3–L4, and L4–L5 segments).

The ROM and FJF for flexion, extension, lateral bending, and axial torsion motion decreased with an increasing weight percentage of PVA in PVAH. When the results of the L1–L2, L2–L3, L3–L4, and L4–L5 segments were averaged, the ROMs of 20 wt% PVAH were lower than those of 3 wt% PVAH by 1.0%, 3.3%, 4.8%, and 1.7% for flexion, extension, lateral bending, and axial torsion motions, respectively. The ROMs of the lumbar segments with 15 wt% and 20 wt% PVAH differed by only 0.45% and 0.86%, respectively, compared with the ROMs and the NP. When the results of the L1–L2, L2–L3, L3–L4, and L4–L5 segments were averaged, the ROMs of 20 wt% PVAH were lower than those of 15 wt% PVAH by 0.5%, 1.6%, 2.0%, and 1.0% for flexion, extension, lateral bending, and axial torsion motions, respectively (Figure 9). The FJFs of 20 wt% PVAH were lower than those of 3 wt% PVAH by 6.5%, 9.2%, and 5.3% for extension, lateral bending, and axial torsion motions, respectively. The FJFs of the lumbar segments with 15 wt% and 20 wt% PVAH differed by only 0.8% and 1.4%, respectively, compared with those of the NP. The FJFs of 20 wt% PVAH were lower than those of 15 wt% PVAH by 2.7%, 3.2%, and 0.7% for extension, lateral bending, and axial torsion motions, respectively (Figure 10). Similarly, the maximum stress values generated in the IAF and OAF surrounding the NP (i.e., NP pressures) decreased with an increasing weight percentage of PVA in PVAH. In the IAF, the maximum stresses of 20 wt% PVAH (average values of the L1–L2 to L4–L5 segments) were lower than those of 3 wt% PVAH by 12.4%, 15.4%, 8.0%, and 12.0% for flexion, extension, lateral bending, and axial torsion motions, respectively. Moreover, in the OAF, the maximum stresses of 20 wt% PVAH were lower than those of 3 wt% PVAH by 12.3%, 1.4%, 14.1%, and 11.8 % for flexion, extension, lateral bending, and axial torsion motions, respectively. The maximum stresses of the AF with 15 wt% and 20 wt% PVAH differed by only 2.1% and 3.2%, respectively, compared with those of the NPs. In the AF, the maximum stress of 20 wt% PVAH was lower than those of 15 wt% PVAH by 6.3%, 4.2%, 4.5%, and 0.7% for flexion, extension, lateral bending, and axial torsion motions, respectively (Figure 11).

## 4. Discussion

This study aims to optimize the composition ratio of PVAH that can reflect the mechanical behavior of the NP and further reflect the range of motion (ROM) and facet joint forces (FJFs) of the L1–L2 to L4–L5 segments for flexion, extension, lateral bending, and axial torsion, although replaced by PVAH. To this end, six types of PVAH with different composition ratios of PVA and PBS were used to replace the NP, and their biphasic material properties were compared with those of the NPs. Furthermore, the ROMs and FJFs of the lumbar segments with PVAH were compared to those of the NP.

The finite element (FE) model of the lumbar spine developed in this study was verified by comparing the current results (ROMs, FJFs, NP pressures) with in vivo experimental data and finite element (FE) analysis results reported in the literature. Although the current results were reasonably consistent with the literature’s findings, previous in vivo experimental results on the ROM for flexion motion exhibited a significant difference from the FE analysis results (Figure 6). This difference was presumably because the experimental measurements on the ROMs were taken with the subjects (11 male volunteers between the ages of 25 and 36 years) standing with their spine maximally flexed [56]. The applied flexion moment and follower load in in vivo experiments could be significantly different from those in the FE analysis. Regarding the FJFs, previous FE analysis results showed large variations depending on the FE model constructed by each researcher, mainly in the shape of the facet joint cartilage and friction conditions applied to the facet joint (Figure 7). These results suggest that the normal lumbar spine FE model proposed in this study reflects the physiological characteristics of the human lumbar spine, which makes it suitable for use in biomechanical analysis.

The stress–strain curves of PVAH obtained from unconfined compression experiments showed a toe region and a linear region and were identical to the compression behavior of various polymer materials (Figure 4) [29,32,34,59]. The Young’s modulus (E) obtained by curve-fitting unconfined compression experimental results using the Holmes–Mow model increased with an increasing weight percentage of PVA in PVAH. This is because the load required for compression increased with the increasing percentage of weight of PVA, owing to an increase in the solid matrix in PVAH. On the other hand, the permeability parameter $k_{0}$ decreased with an increasing weight percentage of PVA; this indicates that the low permeability ($k_{0}$), caused by the small pore size from the high weight percentage of PVA required a long time to achieve steady-state equilibrium in the stress–relaxation behavior. This can be explained by the fact that the smaller pore size of PVAH with lower permeability ($k_{0}$) provides higher fluid flow resistance, preventing fluid from flowing freely within the PVAH [60]. These results are consistent with those of previous studies [61]. The material properties of PVAH obtained by curve-fitting the experimental results were also compared with those of the NP in previous studies. As per the results, E and $k_{0}$ of the NP are between those of 15 wt% and 20 wt% PVAH, respectively, indicating that 15 wt% and 20 wt% PVAH exhibit compressive properties similar to those of the NP.

In terms of dynamic motional analysis of the lumbar segments either with the NP or with PVAH, the ROMs, FJFs, and NP pressures were measured for flexion, extension, lateral bending, and axial torsions. These values of the lumbar segments with NP were similar to those with 15 wt% PVAH and 20 wt% PVAH. The maximum stresses of the IAF and the OAF tended to gradually decrease with an increasing weight percentage of PVA in PVAH for flexion, extension, lateral bending, and axial torsion because the pressures applied by the AF decreased with increasing E of PVAH. This result also suggests that, as the E of the PVAH decreases, the load applied to the AF increases, increasing the risk of causing lesions such as disc herniation due to AF tear. In this study, variations in the ROMs measured from the lumbar segments with different composition ratios of PVA and PBS in PVAH were smaller than variations in the E measured from PVAH with different composition ratios of PVA and PBS. This is considered to be due to the role of the AF, a form that surrounds the NP, which absorbs the shock of the spine during exercise and evenly distributes the pressure applied to the spine to stabilize it [62,63,64,65,66].

## 5. Conclusions

Among the PVAHs used in our study, 15 wt% PVAH was more similar to the NP than 20 wt% PVAH in terms of the ROMs, while 20 wt% PVAH exhibited results more similar to the NP than 15 wt% PVAH in terms of the FJFs and the maximum stresses of the AF. Considering that the FJFs and AF maximum stresses could cause the risk of AF tears and/or the pain in the facet joints, and the difference between the ROMs with 15 wt% and 20 wt% was insignificant, it appears that 20 wt% PVAH is most suitable for replacing the NP. However, it is still possible that PVAH with a composition between 15 wt% and 20 wt% PVAH, such as 17 wt% PVAH may be closer to the properties of the human nucleus pulposus according to the current results. To evaluate PVAH as a functional substitute for NPs, further studies on material properties and fatigue and wear properties for PVAH with compositions between 15 wt% and 20 wt% are needed.

## Figures and Tables

**Table 1:** Material properties of lumbar spine components.

Component	Young’s Modulus (MPa)	Poisson Ratio	Reference
Cortical bone	12,000	0.3	[45,46,47]
Cancellous bone	100	0.2	[45,46,47]
Posterior elements	3500	0.25	[45,46,47]
Cartilage endplate	Holmes–Mow E = 0.5218, υ = 0.38, β = 0.0028, $k_{0}$ = 5.5 × 10^−16^ (m^4^/Ns), M = 0.22		[19]
Bony endplate	1000	0.3	[19]
Cartilage	11	0.4	[48]
Nucleus pulposus	Holmes–Mow E = 0.202, υ = 0.36, β = 1.46, $k_{0}$ = 18.7 × 10^−16^ (m^4^/Ns), M = 4.8		[19]
Inner anterior	Mooney–Rivlin $C_{1}$ = 0.2, $C_{2}$ = 0.01, k = 6 Fiber-pow-linear E = 12.5, β = 5.5, $\lambda_{0}$ = 1.135		[44]
Outer anterior	Mooney–Rivlin $C_{1}$ = 0.2, $C_{2}$ = 0.01, k = 6 Fiber-pow-linear E = 46, β = 4, $\lambda_{0}$ = 1.065		[44]
Ligament	Nonlinear stress–strain curve		[49,50,51]

**Table 2:** Follower load and moment values of lumbar spine movements.

	Follower Load (N)	Moment (N∙m)	References
Flexion	1175	7.5	[52]
Extension	500	7.5	[52]
Lateral bending	700	7.8	[53]
Axial rotation	720	5.5	[54]

**Table 3:** Parameter values used in the Holmes–Mow model for PVAH.

	3 wt% (n = 5)	5 wt% (n = 5)	7 wt% (n = 5)	10 wt% (n = 5)	15 wt% (n = 5)	20 wt% (n = 5)
E (kPa)	2.2±0.5	10.4±3.5	21.0±4.6	52.3±3.6	123.3±21.5	347.4±32.6
υ	0.15±0.5	0.17±0.05	0.22±0.01	0.23±0.03	0.29±0.03	0.3±0.008
β	1.32±0.38	0.46±0.2	0.74±0.16	1.58±0.21	0.46±0.20	0.45±0.25
$k_{0}$ × 10^−16^ (m^4^/N∙s)	23,262±4768	2998±596	357.3±110.7	191.8±84.6	73.8±16.9	11.7±2.2
M	1.6±0.5	5.99±2.11	4.47±2.19	6.83±2.71	8.30±2.25	5.5±2.4
$R^{2}$ (Curve-fitting)	0.928±0.032	0.981±0.019	0.997±0.004	0.999±0.01	0.999±0.000	0.998±0.001

## Data Availability

All relevant data are within the manuscript.
